# A Comprehensive Assessment of the Chinese Version of the Duke Activity Status Index in Patients with Cardiovascular Diseases 

**DOI:** 10.31083/j.rcm2502045

**Published:** 2024-01-29

**Authors:** Yingxue Liao, Haofeng Zhou, Meifeng Liu, Guolin Zhang, Ting Wang, Mingyu Xu, Jiawei Deng, Lan Guo, Huan Ma, Qingshan Geng

**Affiliations:** ^1^Department of Cardiology, Guangdong Cardiovascular Institute, Guangdong Provincial People’s Hospital, Guangdong Academy of Medical Sciences, 510000 Guangzhou, Guangdong, China; ^2^Department of Cardiac Rehabilitation, Huizhou Third People's Hospital, Guangzhou Medical University, 516000 Huizhou, Guangdong, China; ^3^Department of Cardiology, Guangdong Provincial People’s Hospital, Guangdong Academy of Medical Sciences, Southern Medical University, 510000 Guangzhou, Guangdong, China; ^4^Department of Cardiology, Dongguan Hospital of Traditional Chinese Medicine, 523000 Dongguan, Guangdong, China; ^5^School of Medicine, South China University of Technology, 510000 Guangzhou, Guangdong, China; ^6^Department of Cardiology, Dongguan Songshan Lake Central Hospital, 523326 Dongguan, Guangdong, China

**Keywords:** the Duke Activity Status Index, cardiopulmonary exercise testing, exercise capacity, cardiovascular disease, reliability and validity

## Abstract

**Background:**

Exercise capacity serves as a direct representation of cardiac function. The Duke Activity Status Index (DASI), a self-administered 12-item questionnaire, covers aspects of daily living, household tasks, sexual function, and physical activity. Although widely used to evaluate exercise capacity, its validation in Chinese cardiovascular disease (CVD) patients has not been thoroughly explored. Considering the significant cultural and lifestyle differences between China and Western countries, which may influence Chinese patients’ comprehension and responses to DASI, our objective is to culturally adapt DASI for Chinese patients with CVD to ensure its precision in assessing exercise capacity.

**Methods:**

The cultural adaptation of the original DASI questionnaire into Chinese followed a rigorous process to ensure its validity, reliability, and sensitivity to Chinese CVD patients. The study included 107 outpatients diagnosed with CVD who completed the DASI and cardiopulmonary exercise testing (CPET). Cronbach’s alpha, Spearman correlation, and factor analysis were utilized to test reliability and validity. Receiver operating characteristic (ROC) curve analysis was employed to assess the prognostic utility of the DASI.

**Results:**

Participants had a mean DASI score of 39.40 ± 10.75 and a peak oxygen uptake (Peak ${\mathrm{VO}}_{2}$) of 19.53 ± 5.89 mL/min/kg. The Chinese version of the DASI exhibited satisfactory reliability and validity in CVD patients, with a Chronbach’s alpha coefficient of 0.706. The DASI score demonstrated a moderate correlation with Peak ${\mathrm{VO}}_{2}$ measured by CPET (r = 0.67, *p* < 0.001). Factor analysis yielded three factors, accounting for 56.76% of the total variance, with factor 1 contributing to 26.38% of the variance. ROC curve analysis demonstrated that the DASI exhibited discriminative utility in the identification of patients with improved long-term prognosis (*p* < 0.001). The ROC curve had an area of 0.788 [95% confidence interval (CI) = 0.704–0.871]. The DASI score ≥36.85 served as the optimal threshold for enhanced long-term prognosis, exhibiting a sensitivity of 0.80 and a specificity of 0.69.

**Conclusions:**

The culturally adapted DASI questionnaire is a straightforward and efficient tool for reasonably evaluating exercise capacity in Chinese CVD patients.

## 1. Introduction 

Cardiovascular disease (CVD) presents a persistent and significant global health challenge, contributing to substantial morbidity, mortality, and imposing a considerable economic burden on healthcare systems worldwide [1, 2]. In China, the prevalence of CVD continues to surge, with an estimated 330 million current cases, solidifying its position as a primary cause of death in the nation [3, 4]. Effective management of CVD is crucial for improving patient outcomes and reducing the risk of recurrent events. A critical aspect of CVD management is the assessment of cardiopulmonary function and exercise capacity, which are closely related to factors such as treatment outcomes, quality of life, disease progression, and prognosis [5, 6, 7].

Cardiopulmonary exercise testing (CPET) serves as a valuable tool for the comprehensive evaluation of patients’ cardiopulmonary function and exercise capacity [8, 9]. However, its high cost and technical demands limit its applicability, particularly in primary medical institutions. As a result, the development of low-cost, accessible, and easy-to-operate methods for diagnosing and assessing CVD has become increasingly important. In CPET, a commonly used threshold for peak oxygen uptake (Peak ${\mathrm{VO}}_{2}$) to assess prognosis in patients with heart failure is 16 mL/kg/min [10, 11]. Patients with a Peak ${\mathrm{VO}}_{2}$ below this threshold are considered to have a worse prognosis.

The Duke Activity Status Index (DASI) questionnaire, originally developed in English, consists of 12 self-reported items and provides a practical and effective approach for estimating a patient’s functional capacity [12]. These items encompass various daily activities with differing physical exertion levels, including personal care, ambulation, household tasks, and recreational activities. Each item is assigned a specific point based on the energy expenditure required for the activity. Patients indicate whether they can perform each activity without difficulty (“Yes” or “No”), and the total DASI score is calculated by summing the points of all “Yes” items, ranging from 0 to 58.2. Higher score signify better functional capacity. DASI score have been found to correlate with quality of life in heart failure patients [13, 14] and to predict postoperative risk in patients undergoing inpatient noncardiac surgery [15, 16]. Moreover, the DASI questionnaire has demonstrated its prognostic value, as higher score have been associated with improved outcomes and reduced mortality rates in CVD patients [17, 18].

Although the questionnaire has been used in Chinese patient populations [19, 20, 21], its reliability and validity in this specific group have not been thoroughly investigated. Furthermore, in our previous study [20] involving Chinese patients with pulmonary hypertension, we discovered a weak correlation (r = 0.467) between the DASI score obtained from the original questionnaire and Peak ${\mathrm{VO}}_{2}$, a correlation that was even weaker compared to earlier foreign research findings [12, 22]. Notably, during the questionnaire completion process, patients expressed unfamiliarity with certain physical activities listed in the original DASI, such as baseball and bowling, which may contribute to the weak correlation. Moreover, studies examining the relationship between the DASI and Peak ${\mathrm{VO}}_{2}$ in Chinese populations are limited, with previous research utilizing estimated Peak ${\mathrm{VO}}_{2}$ from treadmill tests. Consequently, it is crucial to culturally adapt the DASI questionnaire to ensure its precision in assessing the exercise capacity of the Chinese population.

The primary objective of this investigation is to culturally adapt and validate the DASI questionnaire for Chinese CVD patients. This includes an exploration of the questionnaire’s reliability and validity. Additionally, we will explore whether the adapted questionnaire can effectively identify patients with a better or worse prognosis based on a Peak ${\mathrm{VO}}_{2}$ threshold of 16 mL/kg/min. We hypothesize that the culturally adapted DASI can reflect the participants’ exercise capacity more accurately, as represented by a stronger correlation between the DASI score and Peak ${\mathrm{VO}}_{2}$, and serve as a simple tool to identify CVD patients with better prognosis.

## 2. Materials and Methods

### 2.1 Study Design

This cross-sectional observational study was conducted at Guangdong Provincial People’s Hospital (GDPH), a tertiary-care teaching hospital situated in southern China. The observational protocol of the study obtained ethical approval from the Ethics Committee of Guangdong Provincial People’s Hospital (KY2023-053-02). All procedures were performed in adherence to the principles outlined in the Declaration of Helsinki.

### 2.2 Participants

The study participants were recruited at the cardiovascular clinic of GDPH from March to April 2023. Inclusion criteria were as follows: (1) age ≥18 years; (2) a confirmed diagnosis of CVD in accordance with current guidelines (coronary artery disease, heart failure, valvular disease, and pulmonary arterial hypertension); and (3) ability to complete CPET. Exclusion criteria encompassed instances where participants met any of the following conditions: (1) presence of severe comorbidities, such as unmanaged cardiac conditions or uncontrolled hypertension; (2) inability to engage in effective communication; and (3) unwillingness to sign the consent form for participation.

### 2.3 Procedure

**Fig. 1.**
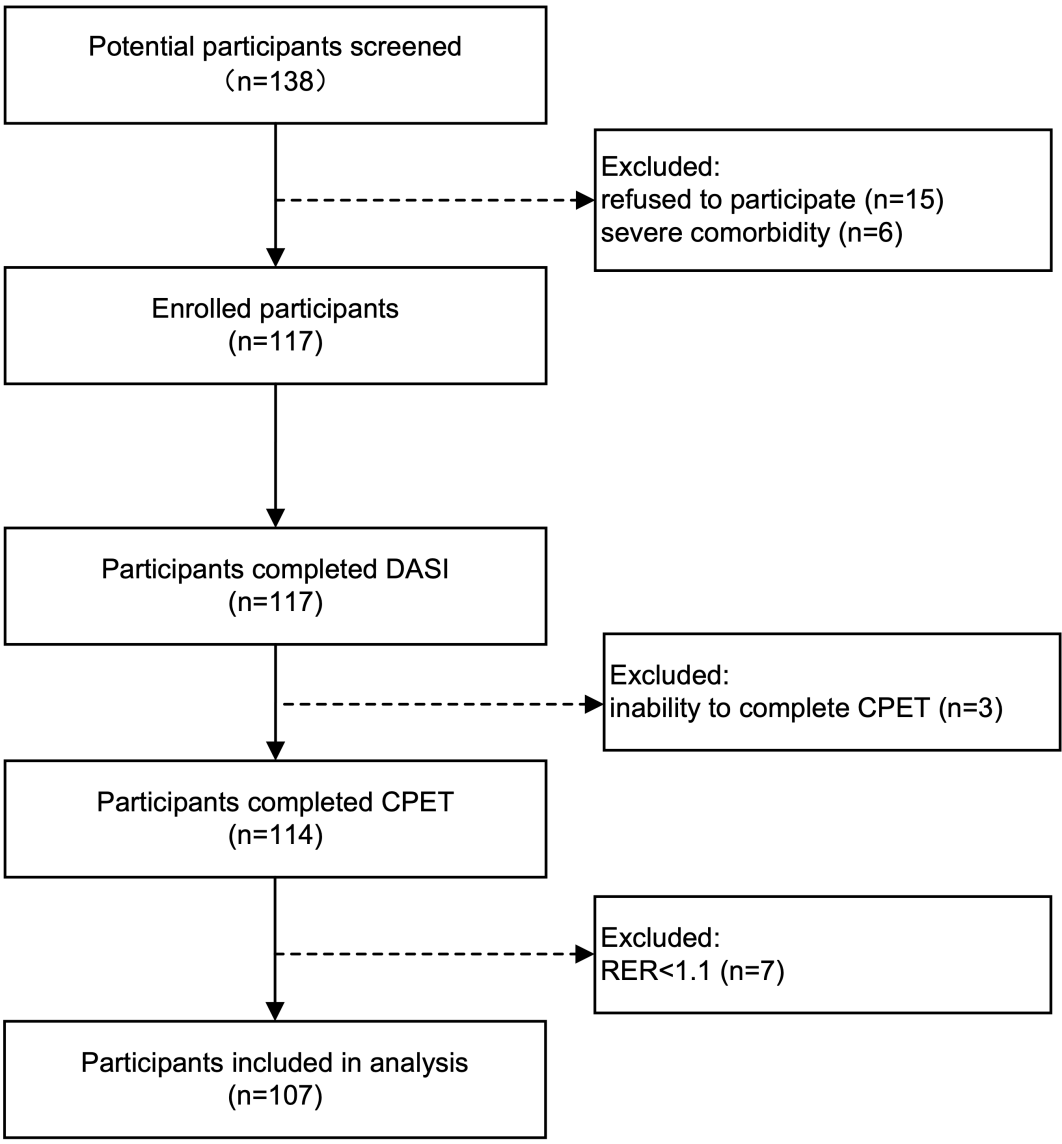
**Study flow diagram**. DASI, The Duke Activity Status Index; CPET, cardiopulmonary exercise testing; RER, respiratory exchange ratio.

The study flow diagram was shown in Fig. 1. All eligible patients were provided with the study introduction and had the opportunity to ask questions about the study before participation. Prior to commencing any study procedures, participants were secured with informed consent. The data collection procedure involved administering the culturally adapted DASI questionnaire first, with the participants’ DASI score being kept blinded to the CPET testing personnel. Subsequently, the participants underwent CPET testing on the same day. Before conducting CPET testing, physicians collected information about the participants’ exercise types, frequency, intensity, and duration to assess their physical activity levels. Additionally, demographic information (age, gender, body mass index [BMI], *etc.*), medical history, and relevant clinical data were obtained through interviews, medical record reviews, and physical examinations conducted by trained research personnel.

### 2.4 Cultural Adaptation and Validation of the Chinese DASI Questionnaire

The cultural adaptation of the original DASI questionnaire into Chinese followed a rigorous process to ensure its validity, reliability, and sensitivity to the Chinese cardiovascular disease patient population [23]. The process began with forward translation by two independent bilingual translators whose native language is Chinese, generating versions T1 and T2. Then, the two translators, a health professional, and a researcher in the field of CVD reviewed the translated questionnaire, resolved discrepancies, and established a consensus version (T-1.2). Subsequently, two different bilingual translators blinded to the original DASI translated the consensus version back into English, resulting in versions BT1 and BT2. Another second expert panel evaluated the conceptual and semantic equivalence between the back-translated English version and the original DASI questionnaire, and necessary modifications were made to the Chinese version. The culturally adapted Chinese DASI questionnaire was then pilot-tested on 15 patients with CVD to assess understandability, acceptability, and any potential issues related to the questionnaire’s content or format. Based on the feedback collected during pilot testing, further revisions were made as necessary. After incorporating the feedback, the final culturally adapted Chinese DASI questionnaire was developed and used in this study. Detailed information on the adaptation process is provided in **Supplementary Table 1**.

### 2.5 Cardiopulmonary Exercise Testing 

CPET was conducted using a cycle ergometer (ERG 910 plus, SCHILLER, Baar, Switzerland) to evaluate aerobic capacity and cardiopulmonary fitness in accordance with established guidelines and practice principles [24]. A calibrated metabolic cart (CARDIOVIT CS-200 Office ErgoSpiro, SCHILLER, Switzerland) was utilized to examine respiratory gas exchange on a breath-by-breath basis, and continuous 12-lead electrocardiogram (ECG) monitoring was performed throughout the test; blood pressure was automatically measured at 2-minute intervals. The CPET protocol utilized a ramp protocol, with participants instructed to maintain a pedal cadence of 55–65 rpm. Physicians used the formula provided by Wasserman *et al*. [25] to calculate the specific incremental work rate based on each participant’s age, sex, height, and weight. Taking into account their physical activity levels, we made a decision on whether to select a power output lower or higher than the computed value, with the aim of achieving a test duration between 8 to 12 minutes. All patients underwent a standardized procedure, which consisted of a 3-minute baseline period and a 3-minute warm-up phase (warm-up, 0 W). Following this, they underwent the incremental exercise test, after which a 5-minute cool-down period was implemented. The test was continued until they presented one of the following termination criteria: (1) achievement of ≥85% predicted maximal heart rate (predicted maximal heart rate = 220-age), (2) plateau in heart rate or oxygen consumption with increasing workload, (3) respiratory exchange ratio (RER) of ≥1.10, (4) rating of perceived exertion (RPE) ≥17 (Borg 6–20 scale); or limiting symptoms were exhibited, including angina, severe fatigue or dyspnea, a decrease in systolic blood pressure with increasing work rate, significant ECG abnormalities, or the patient requested to stop. Peak ${\mathrm{VO}}_{2}$, expressed as mL/kg/min, was determined as the highest 30-second average of ${\mathrm{VO}}_{2}$ achieved during the test. This protocol has been employed in our previous research to assess Peak ${\mathrm{VO}}_{2}$ in both myocardial infarction patients and those with pulmonary hypertension [20, 26].

### 2.6 Sample Size

According to the consensus-based standards for the selection of health measurement instruments (COSMIN), a minimum sample size of 60 patients with CVD was adequate for examining the internal consistency, test-retest reliability, measurement error, and construct validity of the DASI questionnaire [27]. We hypothesized a correlation with a coefficient of at least 0.35 between the DASI score and Peak ${\mathrm{VO}}_{2}$ in CVD patients, considering the reported coefficient of 0.38 in heart failure patients [28]. Therefore, our goal was to enroll a minimum of 85 participants in order to detect this relationship with 90% statistical power, using a two-sided significance level (α) of 0.05, as determined by the PASS software version 15.0.5 (NCSS, Kaysville, UT, USA) tool.

### 2.7 Statistical Analysis

The data analysis was performed using IBM SPSS software version 20 (IBM Corp., Armonk, NY, USA). Continuous variables are presented as mean ± standard deviation, and categorical variables are represented by frequencies and percentages.

The internal consistency was evaluated using Cronbach’s α coefficient. Criterion validity was assessed through the Spearman correlation between the final DASI score and Peak ${\mathrm{VO}}_{2}$ achieved in the exercise test, with the DASI score as the independent variable and Peak ${\mathrm{VO}}_{2}$ as the dependent variable. The strength of the Spearman correlation was interpreted as follows: r = 0.9 to 1.0 indicates a very strong correlation; r = 0.7 to 0.9 signifies a strong correlation; r = 0.5 to 0.7 denotes a moderate correlation; r = 0.3 to 0.5 represents a weak correlation; and r = 0 to 0.3 indicates a very weak or no correlation [29].

Construct validity was examined using factor analysis [30]. A principal components analysis with varimax rotation and Kaiser normalization was conducted. The Kaiser-Meyer-Olkin (KMO) criteria confirmed the adequacy of the correlation matrix, which should be greater than 0.60, and Bartlett’s test with a significance level of 0.05. Factors with eigenvalues greater than or equal to one were considered significant factors. After the rotation matrix, items with a factor loading greater than or equal to 0.5 were included in the factor.

A Peak ${\mathrm{VO}}_{2}$ >16 mL/kg/min was deemed to represent satisfactory functional capacity [31]. To evaluate the discriminative capacity of DASI in differentiating patients with CVD at various risk levels, receiver operating characteristics (ROC) curve analysis along with the area under curve (AUC) calculations were carried out. The AUC values were categorized as follows: AUC = 0.5 indicates no discrimination; 0.7 ≤ AUC < 0.8 signifies acceptable discrimination; 0.8 ≤ AUC < 0.9 denotes excellent discrimination; and AUC >0.9 represents outstanding discrimination [32]. To determine the optimal cut-off value that satisfied the criteria of maximum sensitivity and specificity, we utilized Youden’s index. For subgroup analysis, patients were divided into the high DASI group or the low DASI group according to the established DASI cut-off value. Following this categorization, a *T*-test was employed to compare the two groups in terms of left ventricular ejection fraction (LVEF). No missing values were detected within the primary variables, eliminating the need for imputation procedures. A *p*-value < 0.05 was considered to demonstrate statistical significance for all tests.

## 3. Results

### 3.1 Cross-Cultural Adaptation of DASI into the Chinese Language

Most of the DASI items were translated with minimal cultural adaptation. Three items, however, were adjusted to suit Chinese societal norms. Firstly, in item 9, we replaced “weeding” which is a less common activity in Chinese society, with “gardening or farm work, hoeing” which has a metablic equivalents (METs) value of 3.9 [33, 34, 35], classifying it in the moderate-intensity levels, similar to “weeding”. Secondly, in item 11, “golf, bowling, doubles tennis, or throwing a baseball or football” were replaced by “table tennis, fishing, or doubles badminton”. These replacements were made because these activities are more prevalent in China and are considered to represent moderate-intensity levels, corresponding to metabolic equivalents ranging from 3 to 6 [33, 34, 35]. Lastly, in item number 12, the activities “singles tennis, football, basketball or skiing” were replaced with “singles badminton, mountain climbing” both of which are considered high-intensity activities [35, 36]. During the pilot testing phase, patients encountered no issues in understanding all 12 items and found the activities mentioned to be relevant to their circumstances. As a result, the Chinese version of DASI maintained the principal meanings of the original items, was easy to comprehend, and user-friendly. For the complete Chinese version of the DASI, please refer to **Supplementary Table 2**. Additionally, the English translated version of the Chinese DASI can be found in **Supplementary Table 3**. All participants found the questionnaire easy to respond to. They encountered no difficulties while completing it, demonstrating a clear understanding of all the listed activities.

### 3.2 Participants Physical and Clinical Characteristics

Initially, 138 potential participants were screened for eligibility. Among them, 15 patients declined to participate, and 6 patients with severe comorbidities were excluded. Additionally, 3 patients were unable to complete the CPET due to physical constraints, while 7 others were excluded because of an RER <1.1 in the CPET. Consequently, the analysis included 107 patients. The participant cohort consisted of 60 males (56.1%) and 47 females (43.9%), with a mean age of 48.01 ± 12.22 years, a mean BMI of 22.29 ± 3.22 ${\mathrm{kg/m}}^{2}$, a mean DASI total score of 39.40 ± 10.75, and a mean Peak ${\mathrm{VO}}_{2}$ of 19.53 ± 5.89 mL/kg/min. The physical and clinical attributes of the participants are detailed in Table 1.

**Table 1.** **Baseline demographic and clinical characteristics of the patients**.

Variable		Mean ± SD or n (%)
Age (years)		48.01 ± 12.22
Gender (female/male)		47 (43.9%)/60 (56.1%)
BMI (kg/m^2^)		22.29 ± 3.22
Diagnosis		
	Coronary artery disease	52 (48.6%)
	Valvular disease	20 (18.7%)
	Heart failure	18 (16.8%)
	Pulmonary hypertension	17 (15.9%)
Educational level		
	Primary school	23 (21.5%)
	Middle school	28 (26.2%)
	High school	42 (39.2%)
	College or above	14 (13.1%)
Smoking (yes/no)		28 (22.6%)/79 (73.8%)
Exercise habit (yes/no)		30 (28.0%)/77 (72.0%)
LVEF (%)		53.16 ± 9.43
DASI total score		39.40 ± 10.75
Peak ${\mathrm{VO}}_{2}$ (mL/min/kg)		19.53 ± 5.89
Peak ${\mathrm{VO}}_{2}$ <16 (mL/min/kg)		36 (33.6%)

BMI, body mass index; LVEF, left ventricular ejection fraction; DASI, Duke Activity Status Index; Peak ${\mathrm{VO}}_{2}$, peak oxygen uptake; SD, standard deviation.

### 3.3 Reliability and Validity

For the culturally adapted Chinese version of the DASI, no floor or ceiling effects were observed, as three (3%) patients scored the lowest possible points and eleven (10%) scored the highest. The questionnaire exhibited acceptable internal consistency, as evidenced by a Cronbach’s α of 0.71.

**Fig. 2.**
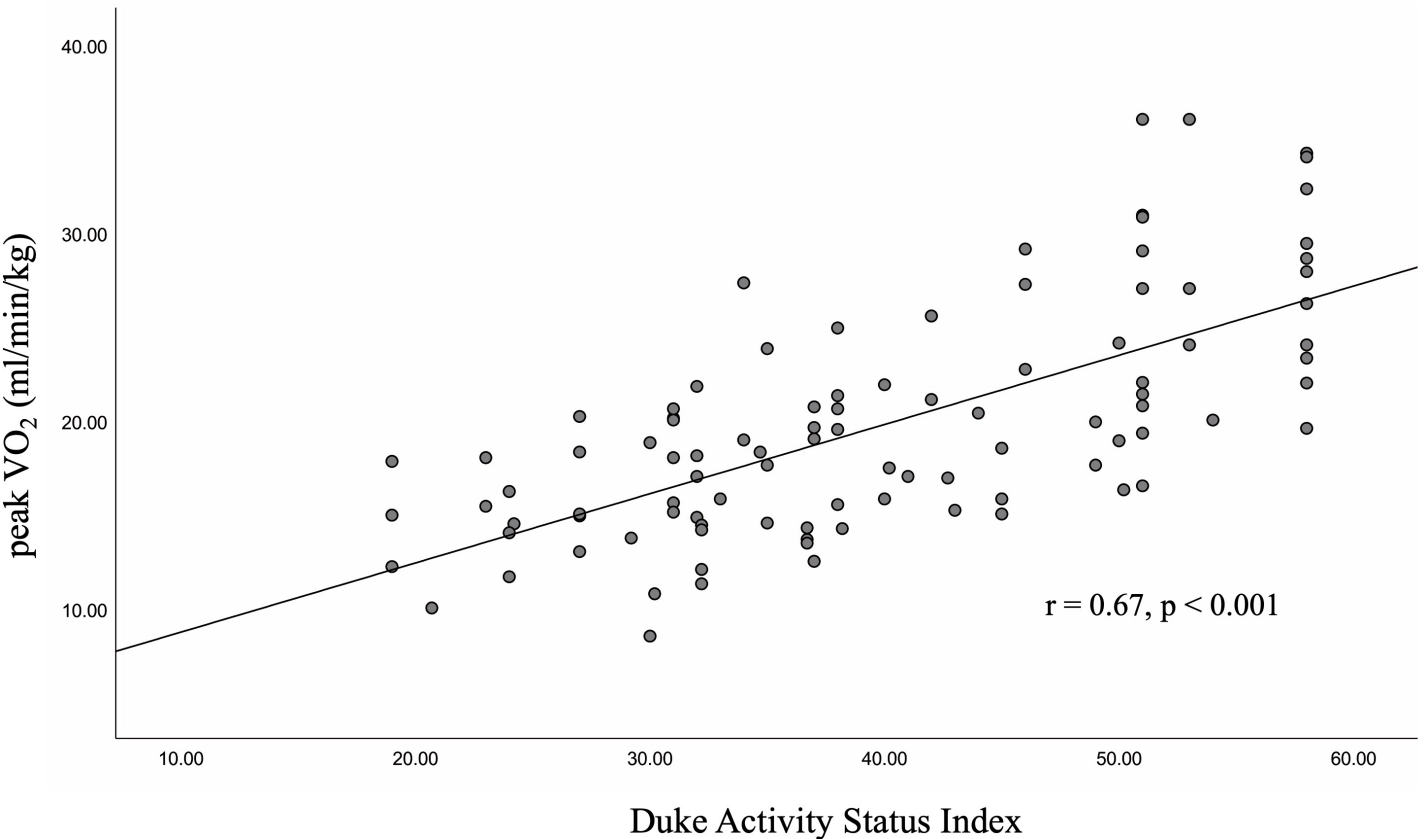
**The Association between the DASI score and Peak ${\mathrm{VO}}_{2}$ in CVD patients**. DASI, Duke Activity Status Index; Peak ${\mathrm{VO}}_{2}$, peak oxygen uptake; CVD, cardiovascular disease.

In assessing criterion validity, a moderate positive correlation was found between the DASI score and Peak ${\mathrm{VO}}_{2}$ (r = 0.67, *p* < 0.001), suggesting that a better functional status correlated with increased exercise capacity among CVD patients. Fig. 2 presents the scatterplot for DASI and Peak ${\mathrm{VO}}_{2}$, complete with the best fit line and 95% confidence interval (CI). A weak correlation was also found between the DASI score and the ${\mathrm{VE/VCO}}_{2}$ (minute ventilation/carbon dioxide production) slope (r = –0.374, *p* < 0.001). The scatterplots for these relationships are shown in **Supplementary Fig. 1**.

To evaluate construct validity, exploratory factor analysis was conducted after excluding item 1 from the questionnaire, as all subjects in the sample reported their ability to perform the proposed activities, resulting in no variance for this particular item. The obtained KMO value (0.71) and Bartlett’s test (*p* < 0.001) indicated the suitability of factor analysis for data processing. A total of three factors were identified, collectively accounting for 56.76% of the total variance, while factor 1 alone explained 26.38% of the variance. Specifically, the first factor comprised items 3, 4, 6, and 7; the second factor included items 5, 9, 10, 11, and 12; and the third factor encompassed items 2, 8, and 9.

### 3.4 DASI as a Predictor of Long-Term Prognosis

**Fig. 3.**
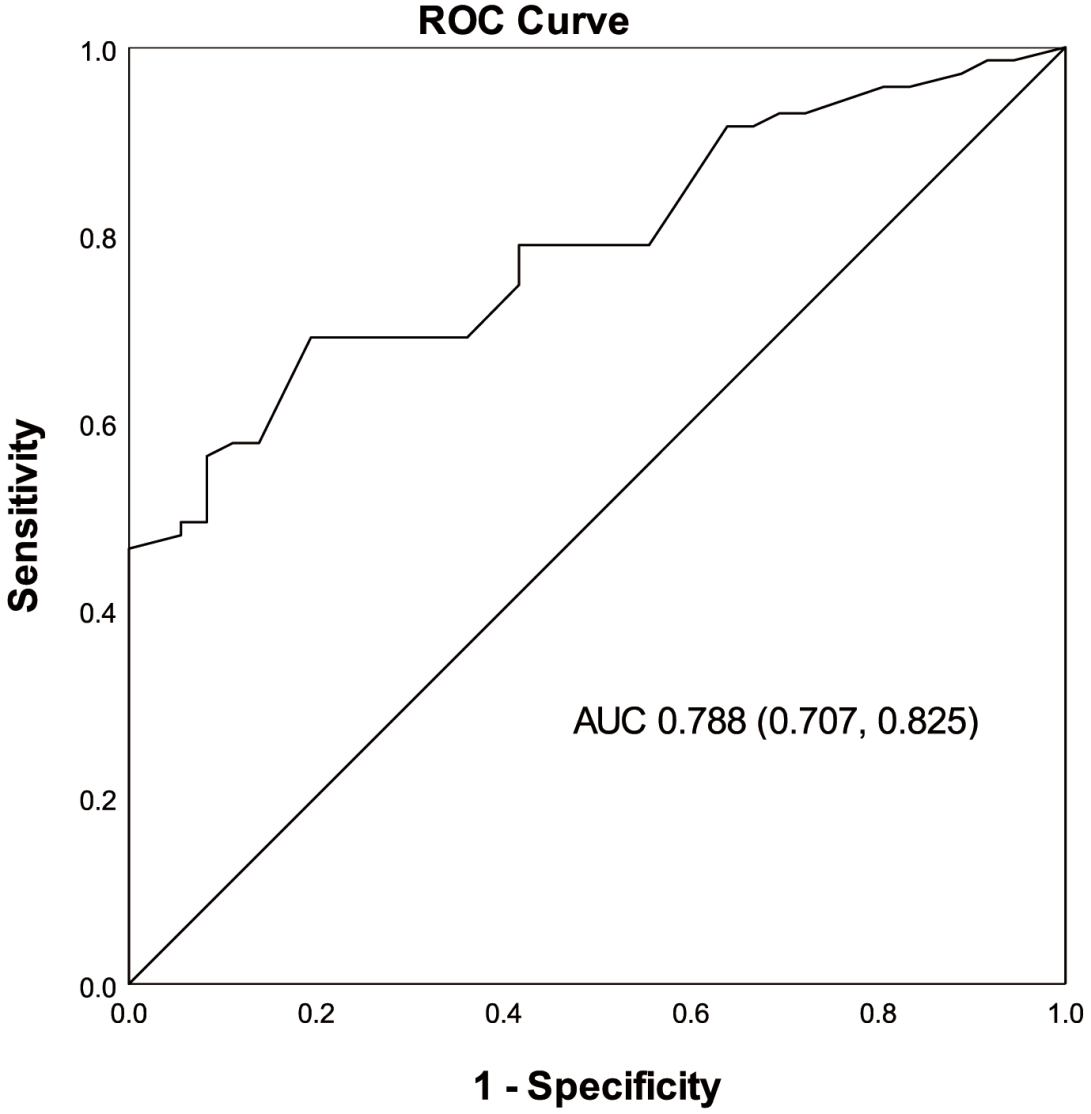
**Discriminative properties of the DASI in identifying the patients with Peak ${\mathrm{VO}}_{2}$ >16 mL/kg/min**. DASI, Duke Activity Status Index; Peak ${\mathrm{VO}}_{2}$, peak oxygen uptake; ROC, receiver operating characteristic; AUC, area under ROC curve.

The ROC curve analysis demonstrated the discriminative ability of DASI in identifying patients with a more favorable long-term prognosis (*p* < 0.001). With an area under the ROC curve of 0.788 [95% CI = 0.704–0.871], the analysis indicated ‘acceptable’ discrimination (Fig. 3). The optimal cut-off value for DASI in detecting patients with better long-term prognosis was found to be 36.85, exhibiting a sensitivity of 0.80 and a specificity of 0.69.

### 3.5 Validation of the Cut-Off Value for DASI

**Fig. 4.**
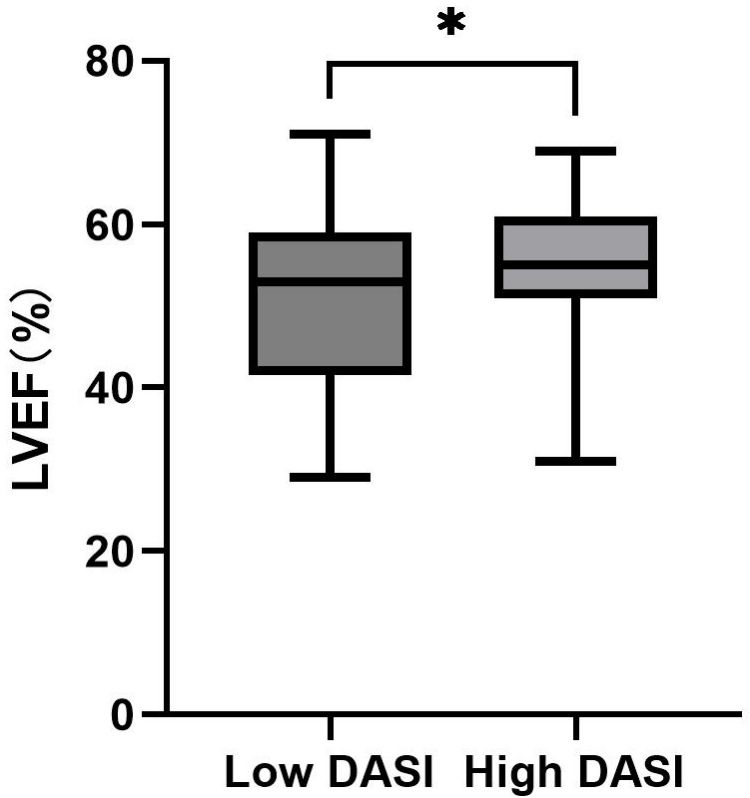
**Comparison of LVEF between High DASI Group and Low DASI Group**. LVEF, left ventricular ejection fraction; DASI, Duke Activity Status Index; *, *p* < 0.05.

Using the established DASI cut-off value of 36.85, we categorized the participants, assigning 71 patients to the high DASI group and 36 patients to the low DASI group. Analyzing the LVEF between these groups, we found that patients with a high DASI score demonstrated significantly higher LVEF compared to those with a DASI score <36.85 (*p* = 0.039). This result supports the credibility of the score 36.85 as an effective prognostic cut-off value, as depicted in Fig. 4.

## 4. Discussion

Our findings demonstrate that the culturally adapted DASI questionnaire possesses acceptable reliability, validity, and the ability to predict long-term prognosis in Chinese CVD patients. These results support that the culturally adapted DASI for the Chinese population can provide a reasonably accurate reflection of the exercise capacity of Chinese CVD patients, endorsing the use of DASI as a valuable assessment tool for this patient population.

The reliability of the culturally adapted Chinese version of the DASI questionnaire was found to be consistent with those reported in other populations [12, 37, 38], suggesting that the cultural adaptation process successfully maintained the questionnaire’s psychometric properties. Concerning construct validity, our exploratory factor analysis identified three factors that collectively accounted for more than half of the total variance, differing from the two factors typically found in most validation studies [14, 38]. These factors represent different dimensions of functional capacity, reflecting the original design intention of the DASI questionnaire. In our study, the factor loadings of item 9 showed a relatively equal contribution to both factor 2 (0.522) and factor 3 (0.534). This indicates that item 9 is incapable of distinguishing between various levels of metabolic cost in physical activities, a situation also noted in the Brazilian version of the DASI [38]. In that study, item 9 was assigned a value of 4 METs during development, but the analysis results showed its correlation with higher METs activities. This could stem from the notable prevalence of urban residents among our study participants. The gardening or farm work activities described in item 9 are activities that are not commonly practiced by urban residents in China. Nevertheless, we have chosen to retain this item due to China’s identity as an agrarian nation, where a substantial proportion of the population resides in rural areas.

In the present study, we observed a moderate positive correlation between DASI score and Peak ${\mathrm{VO}}_{2}$, consistent with previous studies [12, 38]. This correlation is stronger than the weak correlation (r = 0.467, *p* < 0.001) we found in our previous study using the original DASI questionnaire [20]. Several key factors may contribute to this enhanced correlation. First, we culturally adapted the DASI questionnaire to better align with the physical activity habits of the Chinese population, making it easier for patients to understand and respond to DASI questions. Second, compared to our previous research, this study included an additional 18 patients. A larger sample size typically enhances the reliability of statistical analyses and facilitates the more accurate detection of correlations.

Notably, this study’s most prominent advantage is the utilization of CPET for assessing Peak ${\mathrm{VO}}_{2}$, which offers a more accurate and reliable evaluation of exercise capacity compared to other studies that rely solely on the six-minute walk test or estimating Peak ${\mathrm{VO}}_{2}$ [22, 37, 38, 39]. The observed positive correlation between DASI score and Peak ${\mathrm{VO}}_{2}$ supports the criterion validity of the culturally adapted DASI questionnaire and suggests its potential usefulness for healthcare professionals in assessing a patient’s cardiovascular health. To further investigate whether the DASI score could be used to evaluate pulmonary function during exercise, we analyzed the correlation between the DASI score and ${\mathrm{VE/VCO}}_{2}$ slope, an important indicator reflecting pulmonary gas exchange efficiency, disease severity and prognosis [40, 41]. However, the correlation in this study is somewhat weak, suggesting that the DASI score may not be a comprehensive index for evaluating cardiorespiratory function.

When categorizing patients into groups with better or worse long-term prognosis based on the Peak ${\mathrm{VO}}_{2}$ >16 mL/kg/min criterion, our study evaluated the discriminative ability of the DASI in differentiating between these two groups. The findings indicated that the DASI had an ‘acceptable’ discriminative ability for identifying CVD patients with a better long-term prognosis, consistent with results from other CVD studies. An optimal cut-off value of 36.85 was identified for a better long-term prognosis, demonstrating satisfactory sensitivity and specificity. Moreover, the difference of LVEF between groups further validates the clinical utility of the cut-off value, considering LVEF is a strong predictor of poor prognosis in patients with CVD [42, 43]. A study conducted by Mustafaoglu *et al*. [44] demonstrated that a DASI score exceeding 26 was associated with a better long-term prognosis in patients with pulmonary hypertension, exhibiting a sensitivity of 0.74 and a specificity of 0.88. Furthermore, in a study investigating the relationship between preoperative DASI score and postoperative prognosis [15], patients with DASI score lower than 34 were found to have an increased risk of postoperative complications, including mortality and myocardial injury. Although these studies demonstrated that lower DASI score are predictors of poor prognosis, the calculated cut-off values for the DASI differ substantially. Our study’s cut-off value of 36.85 is higher than the values obtained in the other two studies. The variation in cut-off values may be attributed to differences in the physical condition of the patient populations. In the study conducted by Mustafaoglu *et al*. [44], the mean distance traversed in the 6-minute walk test and the DASI score were 427.1 m and 27.9 respectively, suggesting a diminished exercise capacity in these patients relative to those in our study. In contrast, Wijeysundera *et al*.’s [15] research revealed that 57% of their participants had a subjective functional capacity assessment falling within the range of 4 to 10 metabolic equivalents, suggesting a moderate level of functional capacity. Their study also reported an average DASI score of 40.5, similar to the DASI score observed in our study’s population. Another factor influencing the variation is the specific criteria used for categorizing prognosis in these studies. It is plausible that the extent of functional impairment would differ across diseases, which could explain the varying cut-off values. The key takeaway, however, is that the DASI can provide a prognostic cut-off value for different diseases, highlighting its clinical utility.

It is important to acknowledge the inherent limitations of the DASI questionnaire, such as the reliance on patients’ self-reporting, which may introduce biases or inaccuracies in the assessment due to factors like memory lapses or social desirability. However, the results of the educational level (Table 1) in this study indicated that patients of varying educational backgrounds can effectively understand and complete the questionnaire, showcasing its suitability across education levels. Furthermore, it offers advantages such as ease of administration, low cost, minimal burden on patients, and a demonstrated correlation with exercise capacity. Therefore, the findings may hold implications for healthcare teams, particularly in primary healthcare settings within the context of China. The adapted DASI questionnaire, to be specific, could play a role in cardiac rehabilitation, assisting healthcare professionals in tailoring individualized exercise programs, monitoring patients’ progress, and evaluating the effectiveness of interventions to improve functional capacity and overall cardiovascular health.

This study has several limitations that warrant consideration. Firstly, the single-center design may limit the generalizability of our findings - conducting multicenter studies could provide more robust evidence. Secondly, as shown in the data scatter plot in Fig. 2, the DASI score is not a perfect measure of exercise capacity. Therefore, when a precise assessment of exercise capacity is necessary, DASI cannot replace exercise testing [12]. Thirdly, being a cross-sectional study, it does not assess the relationship between DASI score and clinical endpoints. Prospective cohort studies could help investigate this relationship. Fourthly, the participant selection may have introduced bias, as some patients were unable to complete the CPET or were excluded for other reasons, and future studies should consider refining screening criteria. Moreover, this study did not assess test-retest reliability or the sensitivity of DASI to functional capacity changes over time, which should be explored in future research.

## 5. Conclusions

This study validates the culturally adapted DASI questionnaire as a straightforward and efficient tool for reasonably evaluating exercise capacity in Chinese CVD patients. This adapted questionnaire demonstrated satisfactory reliability and validity in this patient group, as well as the ability to discern patients with a better long-term prognosis, thus assisting in the identification of high-risk CVD patients.

## Data Availability

The data presented in this study are available on request from the corresponding author.

## Abbreviations

CVD, cardiovascular disease; CPET, cardiopulmonary exercise testing; Peak ${\mathrm{VO}}_{2}$, peak oxygen uptake; DASI, Duke Activity Status Index; BMI, body mass index; ECG, electrocardiogram; ROC, receiver operating characteristics; AUC, area under ROC curve; RER, respiratory exchange ratio; RPE, rating of perceived exertion; LVEF, left ventricular ejection fraction; METs , metablic equivalents.

## Supplementary Material

Supplementary material associated with this article can be found, in the online version, at https://doi.org/10.31083/j.rcm2502045.
