# Supplementary material for: A Comprehensive Assessment of the Chinese Version of the Duke Activity Status Index in Patients with Cardiovascular Diseases
Source: Rev Cardiovasc Med. 2024 Jan 29;25(2):45. doi: 10.31083/j.rcm2502045 (PMC11263151; doi:10.31083/j.rcm2502045)
Supplement: Supplementary file 1 [file 2153-8174-25-2-045-s1.docx]

Supplementary Table 1. Cultural Adaptation Process of the Duke Activity Status Index (DASI) into Chinese

| Original Version | Versions T1 and T2 | Version (T-1.2) | Back-Translated Version |
| --- | --- | --- | --- |
| 1. Can you take care of yourself, that is, eat, dress, bathe or use the toilet? | T1 你能自己照顾自己吗？比如吃饭、穿衣、洗澡或上厕所。 | 你能照顾自己吗？比如进食、穿衣、洗澡或上厕所。 | BT1 Can you take care of yourself, such as eating, dressing, bathing, or using the toilet? |
|  | T2 你能照顾自己吗？比如，进食、穿衣、洗澡或上厕所。 |  | BT2 Can you take care of yourself, for example, eating, dressing, bathing, or using the toilet? |
| 2. Can you walk indoors, such as around your house? | T1 你能在室内行走吗？比如在家里走动。 | 你能在家里走动吗？ | BT1 Can you walk indoors, like moving around your house? |
|  | T2 你能在家里走动吗？ |  | BT2 Can you ambulate indoors, within your residence? |
| 3. Can you walk a block or two on level ground? | T1 你能在平地上走一两个街区吗？ | 你能在平地上行走一到两个街区吗？ | BT1 Can you walk one or two blocks on level ground? |
|  | T2 你能在平地上行走一到两个街区吗？ |  | BT2 Can you walk one to two blocks on flat terrain? |
| 4. Can you climb a flight of stairs or walk up a hill? | T1 你能爬一层楼梯或一个小斜坡吗？ | 你能爬一层楼梯或在坡地上行走吗？ | BT1 Can you ascend stairs or walk on an incline? |
|  | T2 你能爬楼梯或者在坡地上行走吗？ |  | BT2 Can you climb stairs or walk on a slight slope? |
| 5. Can you run a short distance? | T1 你能跑一小段路吗？ | 你能跑一小段距离吗？ | BT1 Can you run a short distance? |
|  | T2 你能跑一小段距离吗? |  | BT2 Can you run a short distance? |
| 6. Can you do light work around the house like dusting or washing dishes? | T1 你能在家里做轻松的家务吗？比如擦灰或洗碗。 | 你能在家里做一些轻松的家务吗？比如擦灰尘或洗碗。 | BT1 Can you perform light household chores, like dusting or washing dishes? |
|  | T2 你能在家里做一些轻松的家务吗？比如擦灰尘或者洗碗。 |  | BT2 Can you do some light household chores, like dusting or washing dishes? |
| 7.Can you do moderate work around the house like vacuuming, sweeping floors or carrying groceries? | T1 你能在家里做中等体力的活吗？比如吸尘、扫地或拎东西。 | 你能做一些适中的家务吗？比如吸尘、扫地或者提杂货。 | BT1 Can you do some moderate housework, like vacuuming, sweeping, or carrying groceries? |
|  | T2 你能做一些适中的家务吗？比如吸尘、扫地或者提杂货。 |  | BT2 Can you perform moderate household tasks, such as vacuuming, sweeping, or carrying groceries? |
| 8. Can you do heavy work around the house like scrubbing floors or lifting or moving heavy furniture? | T1 你能在家里做重体力活吗？比如擦地板或搬运重家具。 | 你能做重体力的家务吗？比如擦地板、搬运或者移动重家具。 | BT1 Can you do heavy housework, like scrubbing floors or lifting/moving heavy furniture? |
|  | T2 你能做重体力的家务吗？比如擦地板、搬运或者移动重家具。 |  | BT2 Can you engage in heavy household tasks, like scrubbing floors or lifting/moving heavy furniture? |
| 9.Can you do yard work like raking leaves, weeding or pushing a power mower? | T1 你能做园艺劳动吗？比如扫落叶、除草或推电动割草机。 | 你能做庭院里的劳动吗？比如扫落叶或者用除草机除草。 | BT1 Can you do yard work, like raking leaves or using a power lawnmower? |
|  | T2 你能做院子里的杂活吗？比如扫落叶、或者用除草机除草。 |  | BT2 Can you engage in gardening activities, like raking leaves or operating a motorized lawnmower? |
| 10. Can you have sexual relations? | T1 你能进行性生活吗？ | 你能进行性生活吗？ | BT1 Can you have sexual intercourse? |
|  | T2 你能有性生活吗？ |  | BT2 Can you maintain sexual relations? |
| 11. Can you participate in moderate recreational activities like golf, bowling, dancing, doubles tennis or throwing a baseball or football? | T1 你能参加中等强度的娱乐活动吗？比如高尔夫、保龄球、跳舞、双打网球或扔棒球或橄榄球。 | 你能参加中等强度的娱乐活动吗？比如高尔夫、保龄球、跳舞、双打网球或者投掷棒球或橄榄球。 | BT1 Can you participate in moderate recreational activities, like golf, bowling, dancing, playing doubles tennis, or throwing a baseball or football? |
|  | T2 你能参与一些温和的娱乐活动吗？比如高尔夫、保龄球、跳舞、双打网球或者投掷棒球或橄榄球。 |  | BT2 Can you participate in moderate recreational activities, such as golf, bowling, dancing, playing doubles tennis, or throwing a baseball or football? |
| 12.Can you participate in strenuous sports like swimming, singles tennis, football, basketball or skiing? | T1 你能参加剧烈的运动吗？比如游泳、单打网球、足球、篮球或滑雪。 | 你能参与剧烈的运动吗？比如游泳、单打网球、足球、篮球或者滑雪。 | BT1 Can you participate in vigorous sports, such as swimming, playing singles tennis, football, basketball, or skiing? |
|  | T2 你能参与一些剧烈运动吗？比如游泳、单打网球、足球、篮球或者滑雪。 |  | BT2 Can you engage in strenuous sports, such as swimming, playing singles tennis, football, basketball, or skiing? |

Supplementary Table 2. Complete Chinese Version of the Duke Activity Status Index (DASI)

| 项目 | 活动能力 | 是 | 否 |
| --- | --- | --- | --- |
| 1 | 你能照顾自己吗？例如进食、穿衣、洗澡或者上厕所。 |  |  |
| 2 | 你能在家里自由走动吗？ |  |  |
| 3 | 你能在平路走一到两个街区吗？ |  |  |
| 4 | 你能爬一层楼梯或爬小山坡吗？ |  |  |
| 5 | 你能跑一小段路吗？ |  |  |
| 6 | 你能做一些轻体力的家务活吗？例如做饭、洗碗、擦窗户。 |  |  |
| 7 | 你能做中等体力强度的家务活吗？例如拎日常杂物、扫地或者用吸尘器。 |  |  |
| 8 | 你能做重体力强度的家务活吗？例如擦洗地面、搬动或重家具（30-40kg）。 |  |  |
| 9 | 你能做园艺或者农活吗？例如清扫落叶、锄地、推动割草机。 |  |  |
| 10 | 你能进行性生活吗？ |  |  |
| 11 | 你能参加中等强度的娱乐活动吗？例如乒乓球、钓鱼、跳舞、羽毛球双打。 |  |  |
| 12 | 你能参与剧烈的活动吗？例如游泳、羽毛球单打、爬山、打篮球。 |  |  |

Supplementary Table 3. Translation of the Duke Activity Status Index (DASI) from the Complete Chinese Version

| Items | Activity Capacity | Yes | No |
| --- | --- | --- | --- |
| 1 | Can you take care of yourself, such as eating, dressing, bathing, or using the toilet? |  |  |
| 2 | Can you move freely at home? |  |  |
| 3 | Can you walk one to two blocks on flat ground? |  |  |
| 4 | Can you climb a flight of stairs or a small hill? |  |  |
| 5 | Can you run a short distance? |  |  |
| 6 | Can you do some light household chores, such as cooking, washing dishes, or cleaning windows? |  |  |
| 7 | Can you do moderate-intensity household chores, such as lifting everyday objects, sweeping, or using a vacuum cleaner? |  |  |
| 8 | Can you do heavy-intensity household chores, such as scrubbing floors, moving or lifting heavy furniture (30-40kg)? |  |  |
| 9 | Can you do gardening or farm work, like raking leaves, hoeing, or pushing a lawnmower? |  |  |
| 10 | Can you engage in sexual activity? |  |  |
| 11 | Can you participate in moderate-intensity recreational activities, such as table tennis, fishing, dancing, or doubles badminton? |  |  |
| 12 | Can you participate in vigorous activities, such as swimming, singles badminton, hiking, or playing basketball? |  |  |


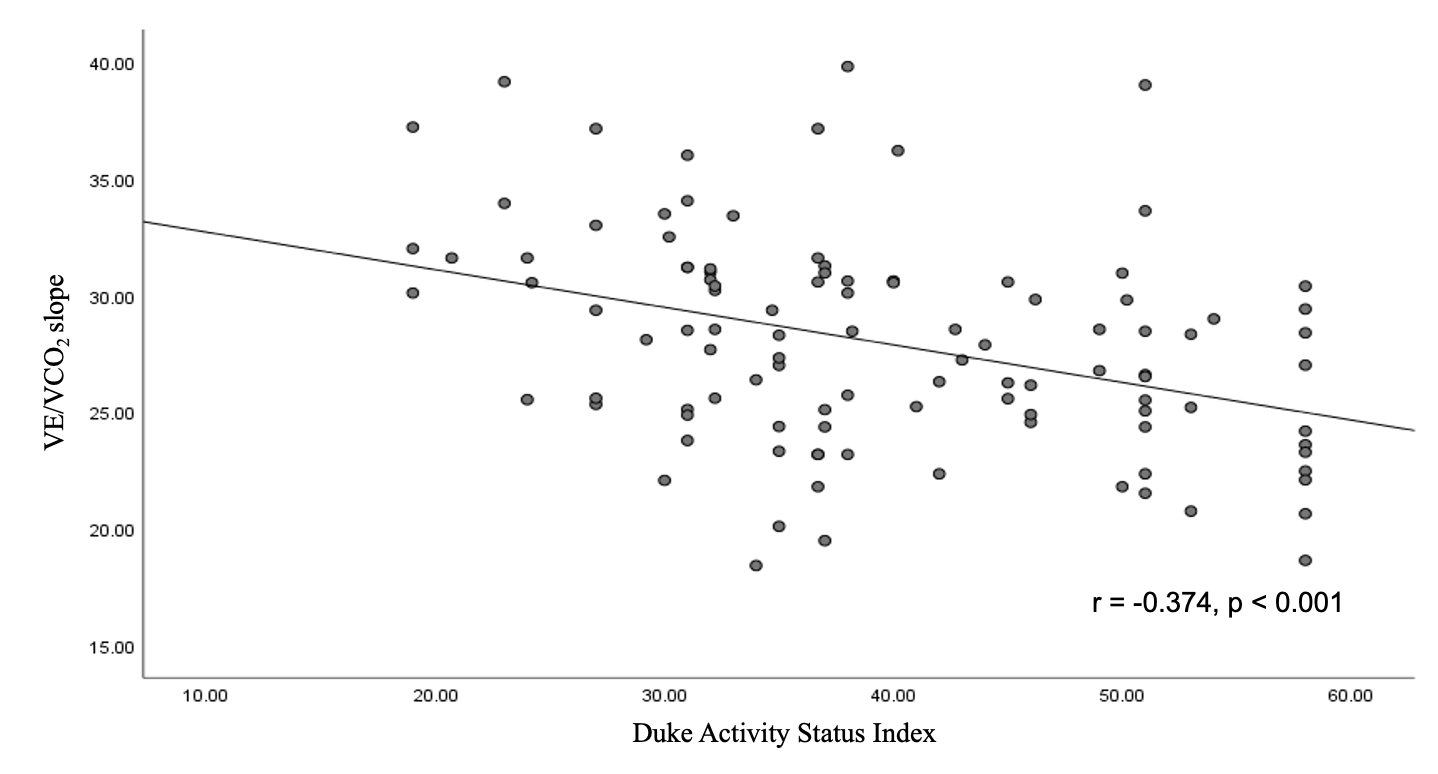


**Supplementary Fig. 1. The Association between the DASI score and VE/VCO_2_ slope in CVD patients.**
